# Excessive Consumption *Hibiscus sabdariffa* L. Increases Inflammation and Blood Pressure in Male Wistar Rats via High Antioxidant Capacity: The Preliminary Findings

**DOI:** 10.3390/cells11182774

**Published:** 2022-09-06

**Authors:** Linaloe Manzano-Pech, Verónica Guarner-Lans, María Elena Soto, Eulises Díaz-Díaz, Sara Caballero-Chacón, Roberto Díaz-Torres, Félix Leao Rodríguez-Fierros, Israel Pérez-Torres

**Affiliations:** 1 Department of Cardiovascular Biomedicine, Institute Nacional de Cardiología Ignacio Chávez, Juan Badiano 1, Sección XVI, Tlalpan, México City 14080, Mexico; 2 Department of Physiology, Institute Nacional de Cardiología Ignacio Chávez, Juan Badiano 1, Sección XVI, Tlalpan, México City 14080, Mexico; 3 Department of Immunology, Instituto Nacional de Cardiología Ignacio Chávez, Juan Badiano 1, Sección XVI, Tlalpan, México City 14080, Mexico; 4 Department of Reproductive Biology, Institute Nacional de Ciencias Médicas y Nutrición Salvador Zubirán, Vasco de Quiroga 15, Sección XVI, Tlalpan, México City 14000, Mexico; 5 Department of Physiology and Pharmacology UNAM, Facultad de Medicina y Veterinaria y Zootecnia, Av. Universidad 3000, Coyoacán 04510, Mexico; 6 Facultad de Estudios Superiores Cuautitlán, UNAM, Km 2.5 Carretera Cuatitlán-Teoloyucan, San Sebastián Xhala, Cuatitlán Izcalli 54714, Mexico; 7 Laboratorio de Patología Veterinaria, Facultad de Ciencias Naturales, Universidad Autónoma de Querétaro, Santiago de Querétaro 76230, Mexico

**Keywords:** proinflammatory interleukins, hypertension, *Hibiscus sabdariffa* L., oxidative stress

## Abstract

*Hibiscus sabdariffa* L. (HSL) has high amounts of antioxidants and many beneficial effects in several pathologies. However, few studies describe the possible harmful effects of high concentrations of HSL. Here we evaluate the effect of excessive and chronic consumption of infusions with different percentages of HSL on some oxidative stress markers in serum, and the possible association with inflammation and increased systolic blood pressure (SBP), in healthy rats. A total of 32 male Wistar rats were used to form 4 groups with 8 animals each. Group 1 control (drinking tap water), group 2, 3 and 4, drinking water supplemented with 15, 30 and 60 g/L of HSL calyxes respectively. SBP was evaluated and determinations in serum of the NO_3_^−^/NO_2_^−^ ratio, glutathione (GSH), total antioxidant capacity (TAC), selenium (Se), TNF-α, IL-1α/IL-1F1, IL-1β, IL-10, extracellular superoxide dismutase (EcSOD), thioredoxin reductase (TrxR) and glutathione peroxidase (GPx) activities, were evaluated. The SBP (*p* = 0.01), GPx activity, GSH, TAC, Se, TNF-α and EcSOD activities (*p* ≤ 0.001) and IL-1α/IL-1F1, IL-1β, TrxR and NO_3_^−^/NO_2_^−^ (*p* ≤ 0.05), were increased but IL-10 (*p* < 0.001) was decreased in rats that consumed the 3 and 6% HSL infusions. The excessive and chronic consumption of HSL may increase the TAC that could lead to a proinflammatory state which is associated with hypertension.

## 1. Introduction

The antioxidant defense systems are critical for maintaining health in biological subjects and they are constituted by enzymatic and non-enzymatic antioxidant components. These defenses are in continuous demand of reducing equivalents provided by glutathione (GSH) and of exogenous compounds including vitamins, amino acids, carotenoids, flavonoids and polyphenols that contribute to counteract oxidative stress (OS) [1]. However, high concentrations and chronic consumption of these agents may lead to prooxidant effects. Nevertheless, few studies have highlighted the prooxidant activity of polyphenols such as catechins, quercetin, epigallocarechin-3-gallate and gallic acid. Excessive amounts of these compounds are associated with breaks in the DNA strand, cytotoxicity, cancer and apoptosis in isolated mitochondria and cultured cells [2].

Different plants constitute a source of natural antioxidants among which *Hibiscus sabdariffa* L. (HSL), also known as Jamaica flower in México, is included. The ingestion of this plant can supply antioxidants such as polyphenols and flavonoids [3]. HSL is commonly consumed in cold and hot beverages and as a diet supplement in different countries. Its consumption is associated with potential health benefits, since it lowers blood pressure, decreases obesity, hypertriglyceridemia, hypercholesterolemia, hyperglycemia, insulin resistance, hyperinsulinemia and LDL oxidation. It also fights cancer, reduces OS, and modulates the immune response [3]. These beneficial properties of low doses of HSL have been reported in different models and several pathologies including metabolic syndrome (MS), spontaneous hypertension and hypercholesterolemia among others [4,5] and in cell cultures exposed to different insults [6].

However, few studies have described the possible harmful effects of HSL at high concentrations. In this sense, continuous consumption of roselle calyces in Wistar Kyoto rats treated with 500 and 1000 mg kg^−1^ led to an increase in the uric acid level [7]. Another study in workers from Nigeria that consumed an HSL infusion containing 2.3 and 4.6 g/kg/day for 12 weeks, showed histological distortion of tubules, disruption of normal testicular epithelial organization, disintegration of sperm cells and a decrease in epididymis sperm counts [8]. In a MS rat model caused by chronic consumption of 30% sucrose in drinking water and that course with OS, a 3% HSL infusion increased the activities the superoxide dismutase (SOD), catalase, glutathione peroxidase (GPx) and the total antioxidant capacity (TAC) but decreased lipoperoxidation, and these changes were associated with a decrease in OS. However, it also increased the activities of some antioxidant enzymes, and the TAC was also increased in the control group in this study [9]. These evidence suggest that the infusion of HSL at low concentrations can be used as an alternative medicinal procedure to improve several pathologies, due to the large amount of antioxidants that it provides; however, chronic and excessive consumption in clinically healthy humans and animals may have adverse and deleterious effects that remain unknown until now.

Therefore, the objective of this study was to evaluate the effect of excessive consumption of HSL infusions on some OS markers in serum and its possible association with inflammation and increased systolic blood pressure (SBP) in healthy rats.

## 2. Materials and Methods

### 2.1. Animals

Thirty-two healthy male Wistar rats weighing between 250–300 g were used to form 4 groups with 8 animals each. Group 1 control (drinking only tap water), group 2, 3 and 4, drinking water supplemented with HSL at 1.5, 3 and 6 percent, respectively. The animals were kept for 4 weeks under the following conditions: 12-h light/12-dark cycle, environment temperature, and relative humidity between the ranges of 18–26 °C and 40–70%. The Laboratory Animal Care Committee of the National Institute of Cardiology “Ignacio Chávez” in Mexico approved the experiments in animals (protocol INC/CICUAL/011/2019) and experiments were carried out in compliance with the Guide for the Care and use of laboratory animals of the National Institutes of Health (NIH). Commercial rodent feed that contains 6% crude fiber, 4.5% crude fat, 23% crude protein, 2.5% minerals and 8% ash was provided (Labdiet 5008; PMI Nutrition International, Richmond, IN, USA). The infusion of HSL at different percentages was administered in drinking water. The preparation of the HSL infusion was as follows; 15, 30, and 60 g of the HSL calyxes were added to one liter of boiling water in each concentration. The solutions were kept boiling for 10 min, and were then left to cool, and stored at 4 °C until consumption. The HSL infusions were prepared and replaced each week to avoid fermentation.

SBP determinations in the rats were done at the end of the experimental period of 4 weeks using a tail cuff attached to a pneumatic pulse transducer (Narco Bio-Systems Inc., Houston, TX, USA) that sends the signals to a computer equipped with a program for data capture and processing (SIEVART version 0.1). Five measurements per rat were performed as previously described [10]. After overnight fasting, the animals were subjected to euthanasia with a guillotine and their blood was collected in vacutainer tubes and centrifuged for 20 min at 936 g and 4 °C. The serum was separated and stored at −30 °C.

The determinations of polyphenols, total flavonoids, vitamin C, and total anthocyanins in the HSL infusion were made by different techniques as previously reported; vitamin C concentration, total flavonoids, total anthocyanins and polyphenols were determined by the methods of the Jagota, Jia, Lee’s and Sánchez-Rangel, respectively [11,12,13,14]. The HSL infusion at 1.5 % contained; cyaniding-3-glucoside (105.8 ± 19.48 mg/L), quercetin (0.29 ± 0.01 mg/L), polyphenols (0.38 ± 0.02 mM/L) and vitamin C, (0.40 ± 0.01 mg/L). The HSL infusion at 3% contained; cyaniding-3-glucoside (551.4 ± 9.6 mg/L), quercetin (0.97 ± 0.02 mg/L), polyphenols (0.70 ± 0.01 mM/L) and vitamin C, 0.80 ± 0.02 mg/L). The HSL infusion at 6% contained; cyaniding-3-glucoside (603.9 ± 10.2 mg/L), quercetin (1.24 ± 0.01 mg/L), polyphenols (0.78 ± 0.03 mM/L) and vitamin C, (0.91 ± 0.01 mg/L). Table 1 shows the volume of liquid consumed and the corresponding equivalence of the antioxidants that were determined in the infusions.

### 2.2. Determinations of NO_3_^−^/NO_2_^−^ Ratio, Glutathione Levels, TAC and Selenium

For the determination of NO_3_^−^/NO_2_^−^ ratio, 100 µL of the serum were incubated with 5 units of nitrate reductase plus NADPH and measured according to the method reported by Griess [15], determining the absorbance at 540 nm. For the quantification of GSH levels, 100 µL of the serum were used and processed according to the method by Ellman, reading the absorbance at 412 nm [16]. For the TAC determination 100 μL of serum were suspended in 1.5 mL of a reaction mixture composed by 300 mM acetate buffer, 20 mM hexahydrate of ferric chloride, and 10 mM of 2,4,6-Tris-2-pyridil-s-triazine dissolved in 40 mM chlorhydric acid pH 3.6. The absorbance was measured at 593 nm, according to the method described by Benzie and Strain [17]. Selenium (Se) determination was performed using 200 µL of serum according to the method described by Soto et al. and the absorbance was read at 600 nm [18].

### 2.3. Determinations of TrxR and GPx Activities

The TrxR activity was determined indirectly by the amount of DTNB in the presence of NADPH to form 2 moles of TNB, using 100 μL of serum according to method described by Soto et al. [18]. The sample was incubated and monitored at 412 nm for 6 min at 37 °C. The activity of GPx was determined indirectly by the amount of NADPH oxidized and is expressed in μmol of NADPH oxidized/min/mL of serum. For this enzyme activity, 100 μL of serum were used and the measurement was made as previously described [18]. The sample was incubated and monitored at 340 nm for 6 min at 37 °C.

### 2.4. Extracellular Superoxide Dismutase Activity

The extracellular (Ec) activity of SOD was determined by nondenaturing gel electrophoresis and nitro blue tetrazolium (2.45 mM) staining as described by Pérez-Torres et al. [10]. 25 μL of serum were applied to a nondenaturing 10% polyacrylamide gel. The electrophoresis was carried at 120 volts. Purified SOD from bovine erythrocytes with a specific activity of 112 U/mg of protein provided by Sigma-Aldrich, St. Louis, MO, USA was used as a positive control to calculate the activity the enzyme.

### 2.5. Determinations of TNF-α, IL-10, IL-1α/IL-1F1 and IL-1β

The determinations of TNF-α, IL-10, IL-1α/IL-1F1 and IL-1β in serum were made with ELISA kits provided by abcam (Rat TNF-α ELISA Kit. and Cat # ab100785, Rat IL-10 ELISA Kit. Cat # ab214566), (Rat IL-1 alpha/IL-1F1 Quantikine ELISA Kit. Cat # RRA00) provided by R&D systems and IL-1β (Rat IL-1β ELISA Kit. Cat # E-EL-R0012) provide by Elabscience Biotechnology, respectively, and they were measured at a wavelength of 450 nm, using a visible light micro plate reader (Stat Fax 3200 Awareness Technology Palm City, FL, USA), according at the manufacturer’s specifications.

### 2.6. Histology of the Thoracic Aorta

A segment the aorta of each rat was preserved in formalin at 10%. Histological sections were made and stained with Jones methylamine silver technique. The histological sections were analyzed with a Carl Zeiss light microscope (66300 Model) equipped with a 9-megapixel Cool SNAP-Pro digital camera and analyzed with the Sigma Scan Pro 5^®^ program (Systat Software Inc., San Jose, CA, USA).

### 2.7. Statistical Analysis

The Sigma Plot program (SigmaPlot^®^ version 14.5, Jandel Corporation, Systat Software Inc., San Jose, CA, USA) was used for statistical analysis, graphs and the linear regressions according to the Constant Variance Test (Spearman Rank Correlation). The Data are presented as mean ± standard error. Statistical significance was determined with Tukey’s one-way ANOVA and post hoc test. A *p* ≤ 0.05 was considered significant.

## 3. Results

**Figure 1:**
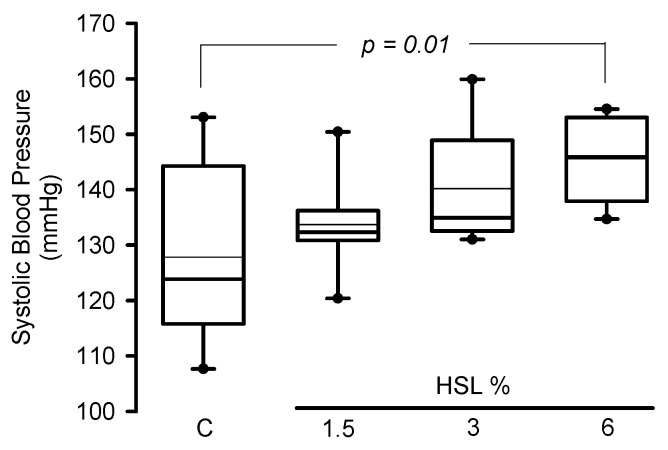
Systolic blood pressure in experimental rats (*n* = 8). The data are presented as median and maximum and minimum ranges (interquartile range 25th percentile, 75th percentile by the type distribution. Additionally, the mean and standard error values of the systolic blood pressure per group expressed in mmHg were as follows: Group 1 = 127.8 ± 5.6, Group 2 = 133.7 ± 2.9, Group 3 = 140.1 ± 3.6, and Group 4 = 145.5 ± 2.6. Abbreviations HSL = *Hibiscus Sabdarrifa* L.

In the rats that consumed the HSL infusion at 3% SBP tended (*p* = 0.09) to increase without reaching a statistically significant difference. However, in the HSL group that received the 6% solution there was a significant elevation when compared to the C group without treatment (*p* = 0.01) (Figure 1).

Table 2 shows the enzymatic activities of TxrR and GPX in the serum from the rats in the experimental groups. Significant increases were present in the HSL group at 6% in comparison with the C group in the TxrR and GPx activities (*p* = 0.05 and *p* < 0.001, respectively). The same table shows that the NO_3_^−^/NO_2_^−^ ratio, GSH levels, TAC and Se, were statistically increased in the groups of rats that consumed HSL infusion at 3% (*p* = 0.05, *p* < 0.001, *p* = 0.01 and *p* = 0.001, respectively) in comparison to C group. The HSL infusion at 6% showed the same tendency as group 3 but with a greater significance in the markers of OS (*p* < 0.001) in comparison with the C group.

Table 3 shows, the inflammation markers in the groups of rats with the different percentages the HSL infusion. The rats that consumed HSL infusion at 1.5% only showed a significant increase in the concentration of TNF-α (*p* = 0.001). However, in the group of rats that was treated with the HSL infusion at 3%, there was a significant increase in TNF-α (*p* = 0.001) and a decrease in IL-10 (*p* = 0.05). In the group of rats that consumed the HSL infusion at 6% there was a significant increase in TNF-α (*p* < 0.001), IL-1β and IL-1α/IL-1F1 (*p* ≤ 0.05), and a decrease in IL-10 in (*p* = 0.001) in comparison with the C group.

**Figure 2:**
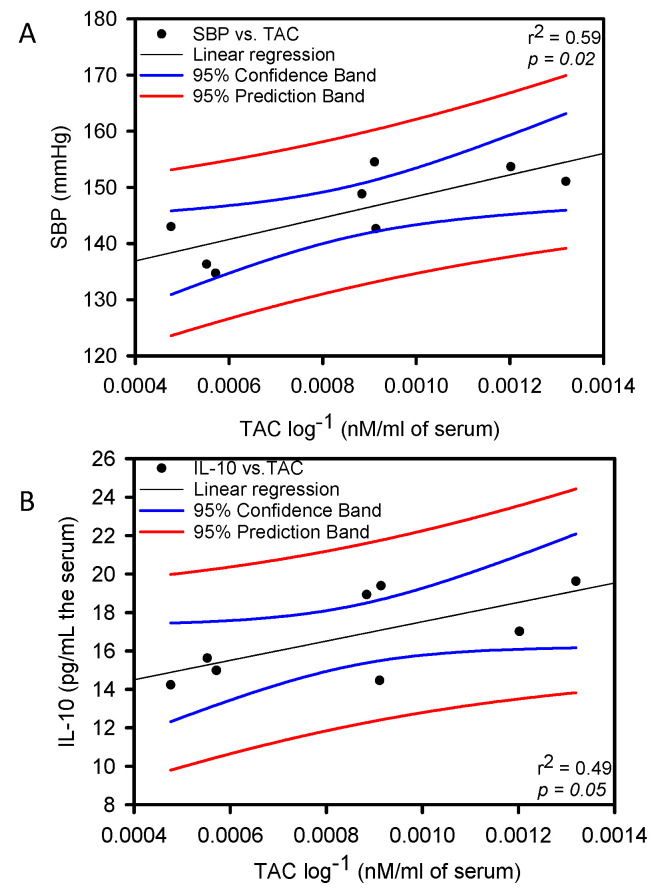
Linear regression between SBP vs. TAC panel (**A**) and IL-10 vs. TAC panel (**B**) Constant Variance Test Spearman Rank Correlation.

Figure 2 shows, that the correlation between IL-10 and TAC was negative with a significant difference (*p* = 0.05 and r^2^ = 0.49). There was a positive correlation with a significant difference between SBP and TAC (*p* = 0.02 and r^2^ = 0.59) in the group of rats that consumed the HSL infusion at 6%.

**Figure 3:**
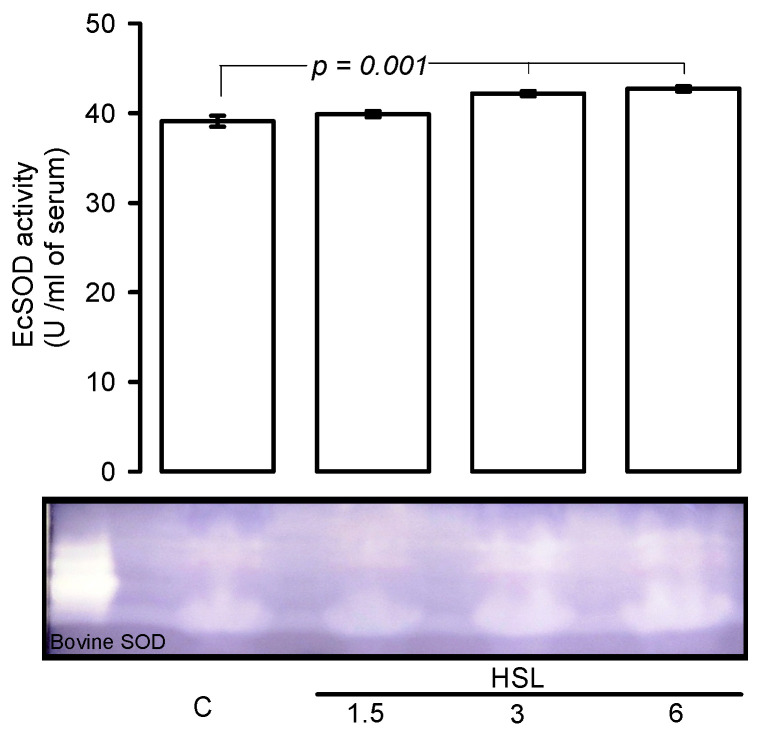
Native gel representative, HSL infusion at 3 and 6% increased the EcSOD activity in serum. In a native gel the TEMED and riboflavin in presence the UV light and oxygen of the medium; produce O_2_^−^, the NBT and EcSOD compete for them, where EcSOD is present; the gel remains transparent, whereas reduced the NBT turns to purple-blue.

Figure 3 shows, that EcSOD activity was significantly increased rats that received the HSL at 3 and 6% (*p* = 0.001) in comparison with C group.

**Figure 4:**
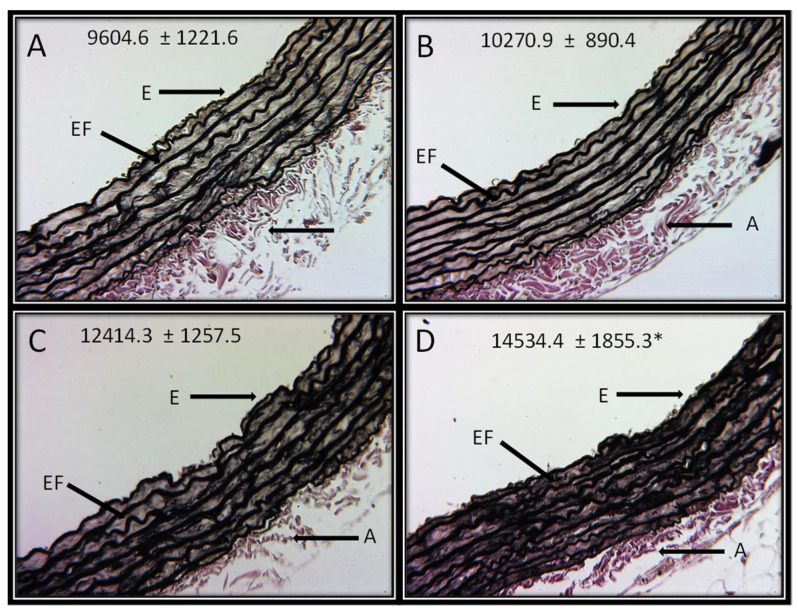
Representative photomicrographs of the histological segments of the aortas of the experimental rats receiving the different percentages of the HSL infusion. The values are expressed as the mean ± SE and units in arbitrary pixels. * C vs. HSL at 6% *p* = 0.04. The histological sections were made and stained by Jones methylamine silver stain technique at 16x. Abbreviations: (**A**) = Control, (**B**) = HSL at 1.5 %, (**C**) = HSL at 3 %, (**D**) = HSL at 6%, E = Endothelium, A = Adventitia, EF = Elastic fibers.

Figure 4A–D shows the representative photomicrographs of the Jones methylamine silver stain in control rats and in animals that received the different concentrations of HSL. It can be observed that the HSL infusion at a percentage of 6% increases in the thickness of the elastic fibers in the thoracic aorta in comparison with the control group. This observation was corroborated with the analysis of the densitophotometry of the photomicrographs, where a greater thickness of the elastic fibers was observed with a significant difference between the same group (*p* = 0.04).

## 4. Discussion

Hundreds of investigations on the antioxidant effects of HSL upon different pathologies that course with OS have been published, and the results are very promising since the ingestion of HSL decreases abnormalities. However, there are few studies that show the toxic effect of the chronic and excessive consumption of high concentrations of HSL. Therefore, the main goal of this study was to evaluate the effect of consumption of infusions with different percentages of HSL on some OS markers in serum of healthy rats and to establish the possible association of this consumption with inflammation and increased SBP. The results show an increase in the SBP of rats that consumed the 6% HSL infusion.

HSL contains tyrosine, and an excess of this amino acid could lead to higher availability of this substrate that is needed for the synthesis of NE by the sympathetic nerve endings. This could contribute to increase the SBP [19]. In addition, in spontaneously hypertensive rats, the consumption the HSL infusion at 1000 mg/kg during 30 to 60 days there was increased mortality in comparison with normotensive rats. In this study, the death cause was attributed to the diuretic property of the solution that may alter the glomerular filtration ratio and may lead to an increase in the uric acid level, serum albumin and deposition of urate crystals in soft tissue and kidney stones [7]. In addition, our results showed a significant increase in the elastic fiber thickness of the aorta. These changes suggest hypertrophy and rigidity that could be reflected in the increase in SBP in the groups of the rats receiving the HSL infusion at 6%. The structural alterations and hypertrophy in the conductive arteries as the aorta has been associated with stiffness, inflammation, and increased SBP [20].

Our results show that in the rats with the treatment with HSL at 6%, there was an increase in the NO_3_^−^/NO_2_^−^ ratio, which constitutes the relation between the secondary metabolites of the oxidation of nitric oxide (NO). The presence of a high ratio is associated with iNOS over-activity or with uncoupled eNOS [1]. Our results showed a positive correlation between the SBP vs. TAC and IL-10 vs. TAC. This suggests that the antioxidants provide by the HSL infusion at 6% increase the TAC and this could impact on the reduction of the anti-inflammatory interleukin IL-10 which is associated with an elevation of SBP. This may be due to the capacity of some antioxidants to autoxidized and generate free radicals. In this sense, the antioxidant excess of agents such as flavonoids and, in particular quercetin [21], may spontaneously oxidize in the presence of transition metal such as iron, copper, Se and aluminum (AL). This oxidation occurs by the Haber-Weiss reaction and may generate reactive oxygen species (ROS) including the hydroxyl radical, H_2_O_2,_ phenoxyl radical, and flavonoid quinone products [22]. These radicals can initiate a cascade of prooxidative events that could lead to a proinflammatory state where iNOS activity is favored. Moreover, agents present in HSL could contribute to a proinflammatory state including kaempferol and quercetin that are two types of lethal mutagens identified in the rosella color by HPLC [23].

On the other hand, our results showed that TNF-α, IL-1α/IL-1F1 ratio and IL-1β were increased but that IL-10 was decreased in the serum of the rats that consumed the HSL infusions at 3 and 6%. This suggests that the excess of antioxidants provided by the HSL infusion could lead to a proinflammatory state that may contribute to increase SBP. Although there was also an increase in TNF-α in the group of rats that consumed the 1.5% HSL infusion, there were no changes in IL-10 in the serum of these rats. This suggests a protective effect against a possible proinflammatory state, which is lost in rats treated with the 3 and 6% infusion of HSL. Furthermore, high doses of prooxidants could lead to production of proinflammatory mediators such as TNF-α and interleukins such as IL-1α, and IL-1β and this could induce nitrosative stress, characterized by increased NO metabolites such as the NO_3_^−^/NO_2_^−^ ratio. In addition, the auto-oxidation process of these antioxidants [24,25] can down- regulate the Akt phosphorylation in a dose dependent manner via flavin oxidase and it may induce ROS generation contributing to the proinflammatory state [26]. Also, inflammation is a key component of endothelial dysfunction that contributes to increase SBP [1].

In addition to the above, HSL can contribute with aluminum (Al) which can be potentially toxic to humans. Al is primarily eliminated from the body via the kidney, and it is excreted in urine and feces. A part of the Al can be retained in the body for several days and another part can accumulate in different tissues. In volunteers that drank an HSL infusion at 2.5% for 16 days, there was a high content the Al in urine and some abnormalities including gastrointestinal problems, diarrhea, nausea and dizziness [27]. Another study showed similar result with respect to gastrointestinal damage. For example, HSL methanol extracts in Swiss Albino Rats showed an increased dose dependent reduction of the intestinal transit and this effect was attributed to quercetin and eugenol [28]. This suggests that high concentration of the HSL infusion could lead to an increased body burden of Al, and that the association with the quercetin and eugenol could contribute to the oxidation of flavonoids [27,28].

Moreover, HSL can provide Se which is an essential nutritional trace element [29]. Several biological functions in the human body depend on the balance of Se levels and its decreased or elevated levels can cause damaging effects. There exists at least 25 Se-enzymes that depend on the concentration of this element including GPx and TrxR. Our results show an increase in this trace element which suggests that the excessive consumption the HSL could lead to selenosis; however, more studies are required to corroborate this hypothesis [2]. In addition, Se may be oxidized in the presence nitrogen species and GSH [30]. In this sense, Se compounds such as selenite and selenium dioxide can react with thiol groups in the presence of GSH to produce O_2_^−^ [30]. This result also suggests that the Se provide by HSL infusion at 3 and 6% could contribute to increase the activities of GPx and TrxR. The increase in the activities de these enzymes could contribute to elevate the TAC. Another study demonstrated that HSL infusion at 3% in control rats increased the GPx activity and TAC in the liver [9]. In addition, EcSOD is a Zn-Cu isoform that is present in serum and is considered as one of the main enzymes for O_2_^−^ detoxification through the dismutation process [31]. The results presented here suggest that the HSL infusion at 3 and 6% increased the EcSOD activity in serum. This increase in its activity may favor the rise of H_2_O_2_, which is the substrate of the GPx, TxrR and catalase and this could contribute to favor the elevation of the activities of the GPx and TrxR, and these three enzymes could contribute to increase the TAC.

In addition, GPx overexpression decreases the formation of thiol groups and H_2_O_2_, and this has been related to reduce signaling from growth factors and deterioration of the mitochondrial function. However, an increase in TrxR, is favored by high Se levels and could restore and reduce the thiol groups, thus leading to a positive feedback process. However, more studies are required to corroborate this hypothesis. In this sense, other studies have shown that the treatment with 10 and 15 HSL doses of 250 mg/kg in Wistar albino rats increase other serum enzymes as the aspartate amino transferase, alanine amino transferases (ALT) and lactate dehydrogenase. These elevations were associated with liver injury, and the high serum level of ALT correlate with liver inflammation [7]. In this sense, a recent study demonstrated that the increase the ALT and gamma-glutamyl tranferase was associate with SBP elevation [32], and another study showed that intra peritoneal administration of epigallocatechin-3-gallate in mice causes liver damage and formation of ROS and this was associated with 100% death of mice [33].

On the other hand, HSL can by itself contribute to increase GSH since it contains cysteine, glycine, and glutamic acid, which are GSH precursors, and could increase the synthesis of this antioxidant [3]. In this sense, our results showed a significant increase in the concentration of GSH in all the groups that received the HSL infusion in this could contribute to the increase of the TAC.

## 5. Conclusions

The excessive and chronic consumption of HSL infusions at 3 and 6% could favors increase the TAC and lead at a proinflammatory state associated to hypertension and with more deleterious effects at a percentage of 6%. However, at low percentages such as 1.5%, the HSL infusion has beneficial antioxidant properties.

### Limitations of the Study

Since our results are preliminary, it is necessary to carry out other studies to reinforce the results, such as the analysis of the liver enzymes aspartate amino transferase, ALT lactate dehydrogenase and gamma-glutamyl transferase in serum. These things considered, the study of the pharmacokinetics of the different percentages the HSL infusions on the activity of the enzymes analyzed in this study would yield interesting results; however, this was not addressed in this study. As future perspectives of this study we could mention that to support these results, it would be necessary to carry out other studies on the impact of the 6% HSL infusion on other organs such as the liver, heart and kidney that could be associated with an increase of the TAC.

## Figures and Tables

**Table 1:** Corresponds equivalence of the antioxidants and the water that consumed per rat/day.

Daily Antioxidant Intake		HSL (%)		
	C	1.5	3	6
Water consumption (mL/day)	50.57 ± 1.90	48.20 ± 1.31	46.38 ± 0.47	47.00 ± 0.75
Cyaniding-3-glucoside (mg/day)	0	5.09 ± 2 × 10^−2^	25.57 ± 4 × 10^−3^	28.38 ± 7 × 10^−3^
Quercetin (mg/day)	0	0.01 ± 1 × 10^−5^	0.04 ± 9 × 10 ^−6^	0.05 ± 7 × 10^−6^
Polyphenols (mM/day)	0	0.01 ± 2 × 10^−5^	0.03 ± 4 × 10^−6^	0.03 ± 2 × 10^−5^
Vitamin C (mg/day)	0	0.01 ± 1 × 10^−5^	0.03 ± 9 × 10^−6^	0.04 ± 7 × 10^−6^

**Table 2:** Oxidative stress markers in serum in the rats of the experimental groups.

Variables (Serum per mL)	Control	HSL (%)		
		1.5	3	6
TAC (nM)	854.73 ± 41.60	1077.97 ± 59.95	1248.48 ± 109.10 *	1405.52 ± 175.92 **
GSH (µM)	1.15 ± 0.02	1.31 ± 0.02 *	1.38 ± 0.03 **	1.42 ± 0.01 **
Se (nM/mL)	0.003 ± 0.0002	0.004 ± 0.0006	0.004 ± 0.0003 *	0.005 ± 0.0003 **
GPx (µmol NADPH oxidized/min)	3.05 ± 0.09	3.12 ± 0.08	3.29 ± 0.05	3.56 ± 0.12 **
TrxR (µmol TNB/min)	0.003 ± 0.0006	0.004 ± 0.0005	0.004 ± 0.0004	0.007 ± 0.001 *
NO_3_^−^/NO_2_^−^ (nM)	2.94 ± 0.25	3.03 ± 0.24	3.69 ± 0.15 *	4.18 ± 0.14 **

* C vs. HSL at 3% or at 6% *p* ≤ 0.05, ** C vs. HSL at 6% *p* < 0.001. Abbreviations: TAC = Total antioxidant capacity, GSH = Glutathione reduced, Se = Selenium, GPx = Glutathione peroxidase, TrxR = Thioredoxin reductase, NO_3_^−^/NO_2_^−^ = Nitrate/Nitrite. NADPH = Nicotinamide adenine dinucleotide phosphate, TNB = Thiobis-2-nitrobenzoic acid. (*n* = 8 animals per group). Values are expressed as the mean ± SE.

**Table 3:** Proinflammatory markers in serum in the rats of the experimental groups.

Variables (Serum pg/mL)	Control	HSL (%)		
		1.5	3	6
TNF-α	143.13 ± 22.13	325.70 ± 46.14 **	297.68 ± 34.85 **	265.81 ± 30.05 **
IL-10	20.27 ± 0.83	19.48 ± 0.84	18.12 ± 0.61 *	16.78 ± 0.8 **
IL-1α/IL-1F1	3858.17 ± 369.28	3862.54 ± 383.19	3771.7 ± 289.50	4903.54 ± 319.82 *
IL-1β	1007.31 ± 14.92	977.52 ± 22.48	1024.60 ± 16.45	1066.89 ± 14.38 *

* C vs. HSL at 3% and 6% *p* ≤ 0.05. ** C vs. HSL at 1.5%, 3% and 6% *p* < 0.001. Abbreviations: TNF-α = Tumor necrosis factor-alpha, IL = Interleukins (*n* = 8 animals per group). Values are expressed as the mean ± SE.

## Data Availability

The datasets generated and analyzed during the current study are available from the corresponding author on reasonable request.
